# The Counteractive Effect of Self-Regulation-Based Interventions on Prior Mental Exertion: A Systematic Review of Randomised Controlled Trials

**DOI:** 10.3390/brainsci12070896

**Published:** 2022-07-08

**Authors:** He Sun, Kim Geok Soh, Samsilah Roslan, Mohd Rozilee Wazir Norjali Wazir, Fang Liu, Zijian Zhao

**Affiliations:** 1 School of Physical Education Institute (Main Campus), Zhengzhou University, Zhengzhou 450001, China; verson.upm@gmail.com; 2 Department of Sport Studies, Faculty of Education Studies, Universiti Putra Malaysia, Selangor 43400, Malaysia; kims@upm.edu.my (K.G.S.); mohdrozilee@upm.edu.my (M.R.W.N.W.); 3 Department of Foundation of Education, Faculty of Education Studies, Universiti Putra Malaysia, Selangor 43400, Malaysia; samsilah@upm.edu.my; 4 Department of Social Work, Zhongyuan University of Technology, Zhengzhou 451191, China; liyuxin2020@gmail.com

**Keywords:** fatigue, mental exertion, ego depletion, self-regulation, intervention

## Abstract

Background: Many investigations have been performed on the effects of mental exertion that consumes self-regulatory resources and then affects physical and/or cognitive performance later on. However, the effect of manipulating self-regulation and interventions to attenuate this negative effect remains unclear. Moreover, there is continuous controversy regarding the resource model of self-regulation. Objective: We conducted a systematic review to assess the literature on manipulating self-regulation based on four ingredients (*standard*, *monitoring*, *strength*, and *motivation*) in order to counter mental exertion and improve physical and/or cognitive performance. The results provide more insight into the resource model. Method: A thorough search was conducted to extract the relevant literature from several databases, as well as Google Scholar, and the sources from the references were included as grey literature. A self-regulation intervention compared to a control condition, a physical and/or cognitive task, and a randomised controlled trial were selected. Result: A total of 39 publications were included. Regarding the four components of self-regulation, the interventions could mainly be divided into the following: (i) *standard*: implementation intervention; (ii) *monitoring*: biofeedback and time monitoring; (iii) *strength*: repeated exercise, mindfulness, nature exposure, and recovery strategies; (iv) *motivation*: autonomy-supportive and monetary incentives. The majority of the interventions led to significant improvement in subsequent self-regulatory performance. In addition, the resource model of self-regulation and attention-restoration theory were the most frequently used theories and supported relevant interventions. Conclusion: In line with the resource model, manipulating the four components of self-regulation can effectively attenuate the negative influence of mental exertion. The conservation proposed in the strength model of self-regulation was supported in the current findings to explain the role of motivation in the self-regulation process. Future studies can focus on attention as the centre of the metaphorical resource in the model.

## 1. Introduction

Self-regulation (SR) is defined as overriding or altering responses, predominantly guided by standards of desirable responses, and includes the regulation of behaviour, thoughts, and emotions [1]. It includes numerous tasks, such as determining which goal to pursue, formulating a strategy for doing so, carrying out the strategy, and protecting the goals from competing concerns [2,3]. For example, setting a goal to exercise in the early morning is an action of SR, as is fighting one’s urge to quit the plan when it is drizzling and windy outside.

The resource model is perhaps the most well-known theory of SR. Baumeister and Heatherton [4] initially proposed the model and hypothesised that regulation relied on a limited or depletable resource, such as strength or energy. Two sets of laboratory experiments [5,6] provided preliminary support for this resource hypothesis. Subsequently, reduced self-regulatory capacity due to mental exertion (i.e., inhibition) was dubbed ego depletion [5]. A meta-analysis [7] confirmed that the basic ego-depletion pattern has been well replicated using a variety of methods. The resource model also proposes that the self-regulatory resource is “global”, which indicates that all SR activities use the same pool of resources. For example, managing an emotional response will have an impact on performance in a completely irrelevant SR-demanding handgrip action [6,8]. Although the self-regulatory resource model [5] has been the most widely used psychological paradigm for addressing the performance decline caused by prior mental exertion, it has been met with several challenges.

Firstly, the nature of the resource has been questioned by numerous scholars. It was suggested that blood glucose may be the metaphorical resource in a study by Gailliot and Baumeister [9]. Even though it had a strong intuitive appeal, this idea has never been proven under scrutiny [10]. In addition, it failed replication in a rigorous test [11].

Secondly, motivation is placed at the centre of self-regulation, such as in the process model of self-regulation [12,13]. Inzlicht et al. argued that performing an initial task (i.e., a have-to task) leads to shifts in motivation. Thus, subjects are less motivated when confronted with another have-to task, dispensing with the idea of the depleted resource. However, subsequent studies have consistently not succeeded in finding evidence of a change in motivation regarding the second task (see [14]). Based on this evidence, Baumeister and Tice [15] argued that resources are different from motivation, and the focus of the resource model was on the expenditure of depletable resources in self-regulation. Thus, it is important to make clear exactly what the metaphorical resource is and the link between the self-regulatory resource and motivation.

Based on the controversy surrounding the theory, the current review recognises that SR encompasses four core ingredients: *standard*, *monitoring*, *strength*, and *motivation* [16]. “Regulation” is a change in order to comply with certain norms, which is challenging without tracking or monitoring present conditions. For example, suppose a soccer player wants to decrease his angry outbursts (*standard*) on the pitch when missing a key scoring opportunity due to a foul by an opponent. In this case, the player must *monitor* his or her behaviours, thoughts, and feelings to curb their display of dissatisfaction in the aftermath of an unfavourable whistle sound. ‘Curbing’ is challenging and requires *strength*, while *motivation* gives the impetus to change. In this instance, if the outbursts lead to a red card, then the player will be punished by suspension for the next match. This situation can boost the soccer player’s drive to change his behaviour.

Notably, Sun and Soh [17] recently conducted a systematic review based on the ingredient of *strength* regarding interventions for mitigating prior mental exertion. The findings of the review supported the strength model of self-regulation from two perspectives: (i) a period of self-regulatory strength-training programs (e.g., 2 weeks of non-dominant hand use) could significantly enhance cognitive or physical performance (e.g., by reducing aggressive behaviour) [18]; (ii) the effects of training programs could be cross-domain. That is, some investigations employed unmatched or unrelated types of tasks (e.g., cognitive vs. physical tasks) between interventions and outcomes and showed dramatic enhancements in performance. However, besides *strength*, it is necessary to obtain a clear picture of the other three ingredients (standard, monitoring, and motivation). In particular, the manipulation of *strength* might occur nor only at the stage of before mental exertion, but also after mental exertion. That is, when fatigue develops after mental exertion is there a method that could be used to recover and enhance the subsequent performance? Moreover, the cross-domain effect should be examined further regarding the “global” hypothesis in the strength model, as suggested by the authors.

Humans must often regulate themselves with a SR resource and consciously utilise mental exertion to attain their highest performance. For instance, to earn high scores in school, students must focus on classes and exert mental effort to overcome potential internal (boredom-induced) or external detractors to their success. The scenario is similar for cyclists who have to use mental exertion to resist the impulse to slow down, even though their body aches [19,20]. Likewise, soccer players have to retrieve and interpret relevant information from complex, competitive environments for a prolonged period [21,22,23], as does a basketball player who tries to shoot three points [24]. Over the past two decades, numerous attempts were undertaken to synthesise the literature, both narratively [25,26] and quantitatively [7,27,28]. These attempts demonstrated negative carryover effects, in which the performance of cognitive activities results in an eventual decline in cognitive and physical performance across a wide range of tasks, including aerobic, resistance, perceptual-cognitive and sport-specific tasks. Nevertheless, none of these reviews (e.g., [21,27,28]) focused on interventions that could counteract the negative carryover effects of the prior mental exertion and benefit subsequent performance. Future studies in this field should focus on effective interventions, not just adverse effects. A recent review summarised all the potential interventions that could be used as counteractive measures [29]. Even though Proost et al. searched the literature with the keyword, “ego depletion”, and included several relevant studies, they did not focus on self-regulation-based interventions that may mitigate mental fatigue while improving cognitive or physical performance. As a result, a comprehensive review of self-regulation-based interventions is necessary.

A growing number of studies examining the effects of mental fatigue on cognitive and physical performance has emerged. Mental fatigue is a psychobiological state that results in feelings of tiredness or lack of energy, caused by prior demanding cognitive activity [30]. Fatigue indicates a decrease in the amount of energy available for future self-regulation; thus, an individual cannot maintain their current effort [14,31]. For example, performing a resource-depleting task (i.e., Stroop task) causing mental fatigue impaired subsequent performance in another resource-consuming soccer decision-making task [32,33]. Therefore, it seems that ego depletion and mental fatigue can be linked together. Moreover, similarly to the investigations examining ego depletion, participants in mental-fatigue studies complete cognitively demanding tasks before performing a cognitive or physical task and are simultaneously compared with a “control” condition. This investigating paradigm is the so-called dual or subsequent task (mental exertion and performance task), which has been used in both study fields [34,35,36].

Relevant evidence can also be found in neuroscience studies. For example, exerting SR is associated with decreased activation in the areas responsible for the effortful control of goal-directed behaviour, including the anterior cingulate cortex, alterations to which are also related to mental fatigue [35]. Similarly, a consistent body of research has demonstrated that inducing mental fatigue through effortful cognitive exertion leads to reductions in activation among the brain regions known to play an important role in regulating effortful behaviour, such the dorsolateral prefrontal cortex, the dorsal anterior cingulate cortex, and the anterior insula [37]. Therefore, it is unsurprising, as Inzlicht and Schmeichel [13] asserted, that “ego depletion is a type of short-term mental fatigue”, and Pattyn and Van Cutsem [38] recognised that research on ego depletion is a seminal contribution to the study of fatigue. 

It is therefore unsurprising that several studies have started to merge this evidence theoretically [39] and practically [27,28]. Notably, many studies showed that the lack of interventions to mitigate mental exertion and enhance performance is the largest limitation of the mental-fatigue research field [21,40]. Therefore, using the resource model to investigate interventions on mental fatigue may give more insight and valuable suggestions to future studies. The current review evaluates all the interventions manipulating these four components of SR and presents a clear overall picture of ego-depletion and mental-fatigue interventions. The results also contribute to and advance the resource model of self-regulation.

## 2. Methodology

**Figure 1:**
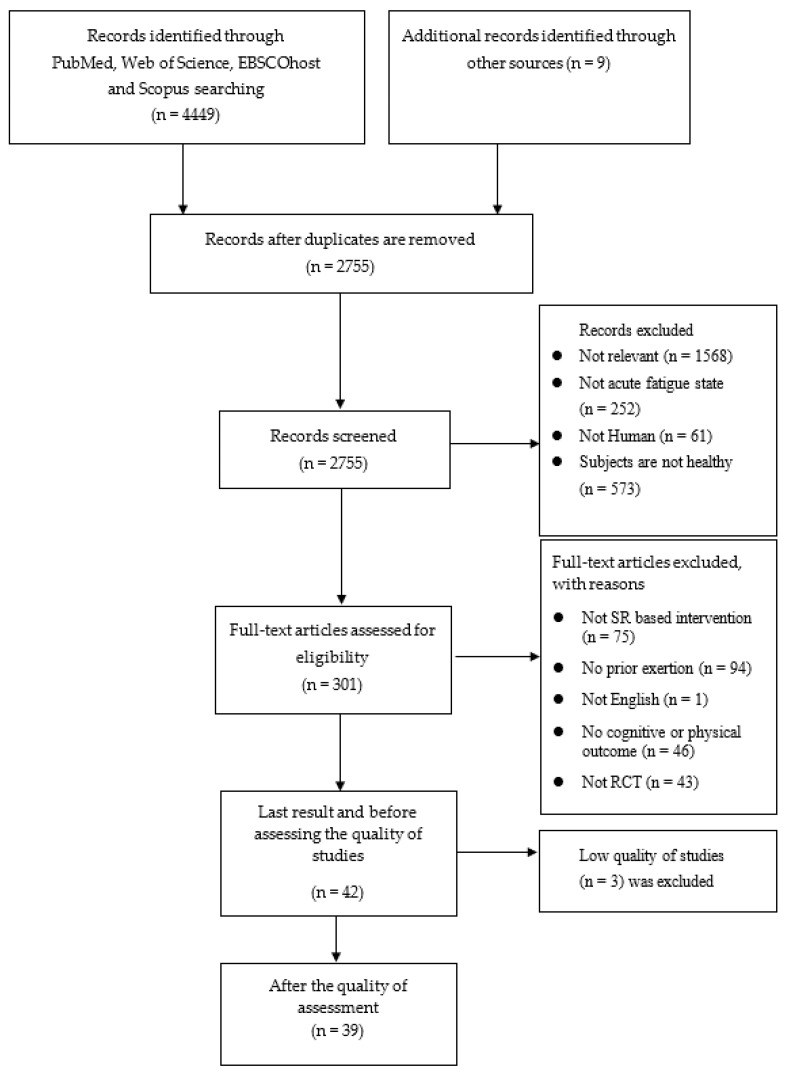
PRISMA of the literature selection.

PRISMA protocol checklist was utilised in the current review [41]. For publications published since 1999, a comprehensive literature search was conducted using the following databases: EBSCOhost (CENTRAL, Psychology and Behavioural Sciences Collection, and SPORTDicus), PubMed, Scopus, and Web of Science (the first intervention based on the strength model of SR was from Muraven et al. (1999)) up to February 2022. EBSCOhost contains many sub-databases. Nevertheless, only three databases were chosen for inclusion because of the relevance of their content: Psychology and Behavioural Science Collection, and CENTRAL (the Cochrane Central Register of Controlled Trials). Furthermore, additional research was found by searching citations and reference lists. Figure 1 illustrates the details of the search results. Expert librarians aided in the data search and confirmed the validity of the search method.

### 2.1. Eligibility Criteria

Using the PICOS method (Table 1), studies were eligible for the review if they: (i) evaluated one of four aspects related to interventions to improve SR in healthy humans with a prior mental exertion task; (ii) included measures of physical and/or cognitive performance; (iii) reported a randomised controlled trial; (iv) were published in English with peer-review process; (v) consisted of records that were published throughout 1999–2022, as the first intervention in the field is from [42].

Moreover, studies that used bioactive substances to counteract mental exertion and improve performance were excluded. The major reason for this was that bioactive substances, such as caffeine, may have strong side effects, such as coronary heart diseases, elevation of cholesterol, or arterial hypertension [43,44]. Although rinsing the mouth with caffeine–maltodextrin can prevent this negative effect and compensate for mental fatigue, it is difficult to use in ecological settings [45].

### 2.2. Search Strategy and Selection of the Literature

Individually and in combination, keywords, truncation, and Boolean operators were utilised to search six databases (Table S1). Furthermore, additional studies that may not have been found in the primary databases were also sought out by focusing on the source of citations of included studies.

Inclusion or exclusion of the articles was created by PICO method (Table 1). In addition, the acute state of mental fatigue or ego depletion induced by prior mental exertion was included, rather than chronic fatigue. A preliminary search was conducted by one reviewer, who reviewed abstracts from 4449 articles eligible for inclusion. A 10% sample of the full abstracts and titles was chosen at random, and the abstracts and titles were individually examined by a second reviewer. After ensuring that there was less than a 5% disparity in the results, a single reviewer performed a screening of all search results. A total of 301 articles were chosen for full-text review after screening. The inclusion of these publications was determined by two independent reviewers. A third reviewer was consulted in the event of discrepancies. 

### 2.3. Protocol and Registration

A PROSPERO (ref. CRD42020210280) record contains the details of the methods and analyses of the current review. While some protocols examine the counteracting effects of mental fatigue regarding the interventions, such as supplements and recovery strategies for SR, none focus comprehensively on the four aspects of SR with regard to physical and cognitive performance concurrently. Therefore, the proposed protocol was recognised on the platform.

### 2.4. Quality Assessment

The quality of the methodology included in this review was assessed by “QuaISyst” [46]. The included studies were assessed with regards to the study design, selection bias, blinding, sample size, analysis, confounders, etc. 

## 3. Results

After the screening, 42 studies met all the eligibility criteria. On assessment, four studies were rated as high-quality, thirty-five as moderate-quality, and three as low-quality. Therefore, 39 publications were included after deleting publications of low quality (Table S2). The most prevalent reason for a quality rating being decreased was a lack of subject blinding, which was difficult to establish in this type of intervention; 36 (85.71%) were downgraded on this basis. The data for a meta-analysis were not pooled because of the heterogeneity in the measurements and interventions. Consequently, only a narrative synthesis of the included studies was developed; it is shown in the Supplementary Materials (Tables S3–S7).

Various research contexts were detected. The effects of an intervention on health-related behaviours [47,48,49], such as improving inhibitions to reject unhealthy food (i.e., high-calorie) and controlling body weight; and exercise or sport performance [50,51,52,53,54,55,56,57], such as increasing the persistence time or total running distance in endurance tests. Several others (Tables S3–S7: context of the study) showed the effect of interventions on the context of social behaviours, such as delaying gratification without impulsive purchase. Moreover, the majority of the participants in the included studies were undergraduates (see Tables S3–S7: subject).

According to the four components of SR, the interventions can mainly be divided into (i) *standard*: implementation of an intervention [58]; (ii) *monitoring*: biofeedback [57] and time monitoring [47]; (iii) *strength*: repeated exercise (cognitive or physical) [8,18,42,48,50,51,52,59,60,61,62,63], mindfulness [53,54,64,65], nature exposure [49,66,67,68,69,70,71,72,73,74,75,76,77], and recovery strategies [55,78]; (iv) *motivation*: autonomy-supportive [56], monetary incentives [79,80,81], and motivational instruction [82].

Moreover, the strength-based interventions (including repeated exercise and nature exposure) seemed to be more reliable than the others in countering the negative effect of prior exertion, as the number of investigations on strength-based interventions far exceeded the others (strength-based intervention: N = 31 vs. the other three types of interventions: N = 8). In addition, the resource model of SR and attention-restoration theory (ART) were the most frequently used theories and supported the relevant interventions (see Tables S3–S7: Intervention-Based Theory).

### 3.1. Interventions Based on the Standard Component

Only one study reported interventions regarding manipulations of the standard aspect. Webb and Sheeran [58] (second experiment) delivered a face-to-face intervention, manipulated implementation intentions, and overcame ego depletion. An implementation intention is subordinate to goal/standard intentions and refers to statements in the following form: “As soon as situation y occurs, I will initiate goal-directed behaviour x” [83,84]. After the mental exertion, the subjects were instructed to form implementation intentions. The results showed that subsequent cognitive performance was significantly improved in the depleted groups when compared with the non-depleted groups in the Stroop task in terms of reaction time (M = 11.62 ± 0.93 vs. M = 13.91 ± 1.23) and error rate (M = 17.15 ± 5.05 vs. M = 27.57 ± 14.71) (Table S3).

### 3.2. Interventions Based on the Monitoring Component

Two studies reported interventions regarding the manipulation of monitoring [47,85]. Monitoring is the process of comparing the current state against the standard/goal to minimise discrepancies [86]. Wan and Sternthal [47] provided accurate time information after a 10-min cross-off-the-letter task. The intervention benefited the cognitive task of puzzle solution regarding the persistence time ($F_{\left(1,\;46\right)}$ = 6.00, *p* < 0.02). In addition, Brown and Bray [57] manipulated the monitoring process after a 10-min Stroop task with biofeedback on heart rate (HR) on a cycle ergometer. The subjects who received the information about their HR could adjust their performance in the endurance task better in terms of total exercise load (*p* = 0.004). Both studies delivered face-to-face interventions.

### 3.3. Interventions Based on the Strength Component

These publications manipulated the strength of SR before or after mental exertion. Specifically, to deliver their intervention, Cranwell and Benford [8] employed smartphones and conducted an internet-based training application. In the first stage, which took place before the mental exertion, 12 studies recruited repeated-exercise training programs to increase self-regulatory strength, including the cognitive and physical domains. Moreover, the durations of the interventions were typically longer than those of the other categories of intervention. The majority of the studies that manipulated the strength component of SR recruited participants to undergo a 2-week repeated-exercise training program, such as posture adjustment [42], modifying verbal mannerism [60], nondominant hand use [18,60], and isometric handgrip exercise [51]. Moreover, an 8-week academic program [59], aerobic exercise [50], and financial monitoring [61] can also improve the strength of SR and reduce vulnerability to mental exertion.

By contrast, there was a study conducted by Miles, Sheeran [63] that showed nonsignificant improvement in handgrip performance after 6 weeks of the repeated exercise training program (i.e., nondominant hand use and Stroop and stop-signal trainings) compared with the control group ($F_{\left(1,\;171\right)}$ = 3.37, *p* = 0.07).

Regarding the recovery stage after mental exertion, several studies focused on interventions regarding mindfulness meditation [53,54,64,65], and more studies investigated exposure to nature [49,66,67,68,69,70,71,73,74,87]. Specifically, Friese and Messner [64] explored short periods of mindfulness meditation, which improved the cognitive and physical performance of the handgrip task significantly (*p* < 0.05). By contrast, Stocker and Englert [53] found no significant improvement ($F_{\left(1,\;30\right)}$ = 0.10, *p* = 0.75) regarding the intervention. To understand these contradictory results, Axelsen and Kirk [65] investigated subjects who were either novices or experienced at mindfulness meditation. The results showed that mental fatigue had the least effect on the SART %NoGo success rate in the experienced mindfulness group, while mental fatigue had a negative impact on performance in both the control group and the novice mindfulness group. Therefore, a background in mindfulness meditation is crucial for the effect. Furthermore, Shaabani and Naderi [54] corroborated that longer doses of mindfulness intervention are more effective at mitigating the effect of prior mental exertion. All the mindfulness studies physically delivered the intervention in a laboratory setting.

Regarding the nature-exposure intervention, four studies were conducted in real outdoor conditions and showed consistent results. In one study, Berman and Jonides [68] instructed subjects to walk in a natural environment for 50–55 min to mitigate the negative effects of 35 min of a backward digit span task (directed attention measure) and successfully compared them with an urban-exposure group (M = 9.40 ± 0.41 vs. M = 8.40 ± 0.33). In addition, Lee and Williams [71], Evensen and Raanaas [72], and Zhang and Kang [75] examined 40 s green roofs, interior plants, and 40 min of nature sounds (birds, water, etc.), respectively. The subjects’ subsequent performance on cognitive tasks improved significantly in these four studies.

Moreover, a milestone study was conducted by Berto [67], who showed that exposure to nature can be conducted virtually. Specifically, Berto used 25 virtual nature photographs as the stimuli and displayed them on a computer. The results showed that directed attention (measured as the reaction time in the cognitive task) significantly increased after viewing the virtual nature photographs on the computer compared with the control group (M = 267.38 ± 73.78 vs. 299.61 ± 41.43). Many studies support this investigation. For example, nature exposure with virtual photographs, improving cognitive performance regarding inhibition, reasoning, and long-term memory significantly [69,70,73]. In particular, Berman and Jonides [68] compared real and virtual nature directly, proving that the number of correct trials on a digit-span task increased in both types of intervention compared to the control groups.

Furthermore, the dose (duration) of the nature-exposure intervention and prior cognitive tasks are crucial to improvement in subsequent tasks (see details in Table S6. Specifically, Laumann and Gärling [66] studied 20 min of nature exposure and found a significant result after 15 min of mental exertion ($F_{\left(1,\;26\right)}$ = 27.52, *p* < 0.001), while Bennett [76] used a 4-min intervention after 15 min of mental exertion; however, Bennett did not find an improvement. In addition, Beute and de Kort [69] found that 3 min of nature exposure after performing a typing task significantly increased cognitive performance in terms of reaction time (M = 1355.56 ± 103.91 vs. M = 1436.87 ± 105.61, $T_{\left(50\right)}$ = 2.7, *p* = 0.009). A 4-min Stroop task added to the prior mental exertion task with the same nature-exposure intervention, on the other hand, did not show any improvement in terms of reaction time (*F* < 0.3, *p* > 0.575) or omission ($H_{\left(3\right)}=4.0,\;p=0.263$).

Overall, three types of intervention aiming at strength recovery received particular attention in the relevant literature: repeated-exercise training programs, mindfulness, and nature exposure. Notably, in addition to Miles and Sheeran [63], other studies showed consistent improvement in self-regulatory strength and subsequent performance. Nature exposure can enhance directed attention and facilitate the recovery of mental exertion; however, the dose (the duration of the intervention) is crucial. Furthermore, the psychological background determines the effect of mindfulness interventions.

### 3.4. Interventions Based on the Motivation Component

Motivation is one important aspect of SR that was manipulated to buffer the effect of prior mental exertion. These types of intervention mainly focused on monetary incentives, including ideal money [79] and real money [80]. Moreover, subjects who receive autonomy-supportive regulation freely can develop inherent satisfaction, which counteracts ego depletion, improving their performance in subsequent endurance trials ($F_{\left(1,\;67\right)}$ = 6.28, *p* < 0.001) [56]. Consistently, subjects who had decreased motivation performed worse in the subsequent task [82].

## 4. Discussion

In the current review, we aimed to assess the literature on the manipulation of SR to mitigate mental exertion and enhance physical and/or cognitive performance. All the investigations were categorised as four aspects regarding SR interventions.

Notably, interventions based on the *strength* component, especially repeated-exercise training programs and nature exposure, were investigated more thoroughly than the others, because the number of these two interventions was far greater than that of the others (see Tables S5 and S6), and the interventions were comprehensively examined according to their type (i.e., repeated training programs: financial monitoring, internet-based application training, nondominant hand using, etc.; nature exposure: real and virtual).

One potential reason that most studies focused on the *strength* component was the higher reliability of the resource model of SR. In the model, *Strength,* also known as energy, is essential, and it can be temporarily depleted during self-regulation [16]. Over the last two decades, the model has been widely utilised to explain performance declines due to prior mental exertion [5,6].

Moreover, the results of the improvements were rather consistent in the *standard*, *monitoring,* and *motivation* components, which can provide partial evidence for the current theory of the resource model regarding SR. The following sections discuss this in more detail.

### 4.1. Interventions Based on the Strength Component

#### 4.1.1. Repeated-Exercise Training Programs

In a manner that is consistent with Sun and Soh [17], the current review also showed that self-regulatory *strength* training programs can significantly enhance cognitive and physical performance after mental exertion (see Table S5). Furthermore, the current review highlights the conservation hypothesis regarding the controversy of the role of motivation in self-regulation. Specifically, we noted that one important challenge proposed that motivation could explain the depletion effect [12]. Briefly, Inzlicht et al. argued that in a standard dual-task paradigm experiment, the first task is typically strenuous, or the participants are unwilling. After performing the task, the participant loses the motivation to maintain their performance on the subsequent task. In other words, the depletion effect is due to a reduction in motivation rather than depleted resources. However, increasing numbers of investigations show inconsistencies. For example, soccer players did not show motivational changes after depleting tasks, despite reductions in intermittent endurance [36] and soccer technique [88]. Xiao and Dang [89] also did not find any indication of changes in motivation in depleted conditions among participants. Thus, the evidence further supports the updated conservation version of the resource model, which is that motivated people may conserve more resources for the subsequent task and maintain their performance [90]. Miles and Sheeran [63] and the other studies shown in Table S5 may have inconsistent results because of this conservation, caused by motivational differences. 

The finding of conservation could be an explanation for why *motivation* has a counteractive effect on depletion/mental exertion (shown in Table S7). However, if the depleted strength is severe enough, could no amount of motivation make up for it? Future studies may investigate this amount more closely, since the current review shows consistent improvement regarding the effects of *motivation* on interventions (see Table S7).

#### 4.1.2. Nature Exposure Interventions

Exposure to nature is defined as “direct physical or sensory contact with the natural environment” (p. 137, [91]), and it positively influences psychological conditions (well-being, mental fatigue, etc.) through perceived restorativeness [92,93]. Notably, all 13 investigations related to nature exposure recruited ART as the theory (see Table S6: nature exposure), and two investigations combined the resource model and ART [69,70].

Following James’s distinction between voluntary and involuntary attention, ART distinguishes between directed attention and involuntary attention. Involuntary attention allows the attentional system to rest and recover, while voluntary attention requires effort and can be exhausting. Therefore, Berto and Baroni [87] argued that ART offers an approach to understanding what is being fatigued or depleted and suggest how this resource can be restored. The effort of attention can be avoided if individuals can surround themselves with enough involuntarily interesting things [94]. These interesting things mainly exist in high-fascination environments, such as scenes of nature [67].

Previous studies only focused on the dual-task paradigm and tried to discover the moderating or mediating variables [13,15], which cannot provide a clear picture, causing continuous controversy regarding the theory of SR. By contrast, the counteractive effects of relevant interventions are highlighted in the current review. If there is a resource that can be limited and depleted among participants, we believe its replenishment would allow us to uncover this mysterious and metaphorical resource. Surprisingly, nature exposure, which is in the environmental psychology field, emerged in the current review, and it was largely ignored by previous studies, which concentrated on the dual-task paradigm.

Thus, particular attention was paid to nature exposure in the current review. Natural scenes attract involuntary attention and replenish directed attention; consequently, participants can perform better in subsequent strenuous tasks. Moreover, there is evidence showing that physical exercise [95,96] and cognitive tasks [97] are largely beneficial for attention, which can support the improvement of SR after repeated-exercise training programs, which are shown in Table S5. Kaplan and Berman [98] used a similar description with the resource model: “the term resource implies that there is something finite in quantity and depleted by heavy demands”. In addition, James argued that this nebulous concept is composed of more concrete processes such as attention. However, most previous studies focused on the depleted resource of SR, which lacked the design features necessary to disentangle the effects of attention from the actual effects of practice (see Tables S3–S7). Although some studies noted that attention may influence the depletion of resources, the attention argument only builds upon the motivational argument. For example, Inzlicht and Schmeichel [12] indicated that depleted participants attended all the more to rewards, rather than task performance. Therefore, several questions are prompted. For instance, what is the relationship between attention and the SR resource? Since attention is manipulated by exposure to nature, it could benefit self-regulatory capacity; future studies could further investigate attention as the centre of the SR.

Sun and Soh [17] corroborated the “global” hypothesis in the strength model of self-regulation from the finding of the cross-domain effect of *strength*-based training programs. The findings of the current review could further explain and prove the cross-domain effect of the other three ingredients of the self-regulation process. Specifically, Table S6 (*strength*-manipulated interventions after mental exertion) shows that attention recovery could improve physical performance, such as the Pland exercise: [53], basketball free shooting [54], and working memory measured in backwards digit span [49]. Since attention (e.g., directed attention) theoretically and practically overlaps with self-regulation [69,98], it was unsurprising to find this cross-domain effect in the current review.

### 4.2. Interventions Based on the Other Three Components

Only eight studies were found that manipulated three components in the SR (i.e., *standard*, *monitoring*, and *motivation*), and the results were consistent. All of the studies showed improvements in subsequent performance (Tables S3, S4 and S7). Therefore, it is unsurprising that a cybernetic model was used to explain self-regulatory behaviour [99]. Carver and Scheier [100] proposed discrepancy-reducing feedback loops and explained the model (see Carver and Scheier, Figure 1). According to the model, when a sense deviates from a comparison value, the perception is compared via a comparator mechanism from an input function. Next, the output function is turned on to reduce the discrepancy. The overall aim is to minimise deviations from the standard of comparison. However, the model only shows the loop, and the maintenance of the loop requires “energy”. The cybernetic model highlights the *monitoring* component; however, the resource model of SR, focuses more on *strength*, which is how much energy/resource is needed to eliminate the discrepancy after finding the different states. This elimination may be controlled by attention and influenced by *motivation.*

## 5. Implications and Future Directions

This review has a few limitations. First, because of the wide range of measurements and training methods used, this review cannot be considered a meta-analysis. Selecting only English-language publications may also have narrowed the scope of the findings.

The current review supports the resource model of SR and shows that it is still the most reliable theory in this academic field. However, the metaphorical resource remains unclear. Attention should be examined more closely in future studies. In particular, in the design, it is necessary to disentangle the effects of attention from the actual effects of practice.

Mental fatigue as a novel topic. It has been investigated particularly thoroughly in sports, such as soccer [101], basketball [24,102], badminton [103], swimming [104], and Australian football [105], showing that it impairs subsequent performance. Future studies could consider further interventions to counter these adverse effects. 

Notably, Martin and Thompson [106] conducted a preliminary investigation into SR that may influence the impact of mental exertion on physical performance (i.e., endurance). Thus, it may be promising for future studies to utilise some interventions from the ego-depletion area shown in Tables S3–S7, which present a more holistic approach to capturing daily SR.

## 6. Conclusions

In conclusion, the findings of the current review support the resource model of SR and show that interventions manipulated using the four components of SR are effective for improving subsequent outcomes. In addition, the conservation hypothesis could explain the role of *motivation* in self-regulation. Future studies could investigate the relationship between different levels of *motivation* and depletion/mental exertion more closely. The cross-domain effect of *strength* training on directed attention in the nature-exposure intervention was highlighted again in the current review. Attention may be the centre of the metaphorical resource in the model and a topic for future studies to investigate.

## Figures and Tables

**Table 1:** The PICOS method for the eligibility criteria (participation, intervention, comparison, outcome, and study design).

PICOS	Details
Participation	Healthy humans
Intervention	One of the four aspects of SR
Comparison	Intervention vs. non-intervention groups
Outcome	Physical and/or cognitive performance
Study design	Randomised controlled trial

## Data Availability

The datasets generated during and/or analyzed during the current study are available from the corresponding author upon reasonable request.

## Supplementary Materials

The following supporting information can be downloaded at: https://www.mdpi.com/article/10.3390/brainsci12070896/s1, Table S1: Detailed search strategy; Table S2: “Qualsyst” of quality assessment; Table S3: Characteristics of standard manipulated interventions details; Table S4: Characteristics of monitoring manipulated interventions details; Table S5: Characteristics of strength manipulated interventions (preventing before mental exertion) details; Table S6: Characteristics of strength manipulated interventions (recover after mental exertion) details; Table S7: Characteristics of motivation manipulated interventions details.

## References

[1] Baumeister R.F. (2014). Self-regulation, ego depletion, and inhibition. Neuropsychologia.

[2] Ludwig R.M., Srivastava S., Berkman E.T. (2019). Predicting exercise with a personality facet: Planfulness and goal achievement. Psychol. Sci..

[3] Fujita K. (2011). On Conceptualizing Self-Control as More Than the Effortful Inhibition of Impulses. Personal. Soc. Psychol. Rev..

[4] Baumeister R.F., Heatherton T.F., Tice D.M. (1994). Losing Control: How and Why People Fail at Self-Regulation.

[5] Baumeister R.F., Bratslavsky E., Muraven M., Tice D.M. (1998). Ego depletion: Is the active self a limited resource?. J. Personal. Soc. Psychol..

[6] Muraven M., Tice D.M., Baumeister R.F. (1998). Self-control as limited resource: Regulatory depletion patterns. J. Personal. Soc. Psychol..

[7] Hagger M.S., Wood C., Stiff C., Chatzisarantis N.L.D. (2010). Ego depletion and the strength model of self-control: A meta-analysis. Psychol. Bull..

[8] Cranwell J., Benford S., Houghton R.J., Golembewksi M., Fischer J.E., Hagger M.S. (2014). CP Increasing self-regulatory energy using an internet-based training application delivered by smartphone technology. Cyberpsychol. Behav. Soc. Netw..

[9] Gailliot M.T., Baumeister R.F. (2007). The physiology of willpower: Linking blood glucose to self-control. Pers. Soc. Psychol. Rev..

[10] Kurzban R. (2010). Does the Brain Consume Additional Glucose during Self-Control Tasks?. Evol. Psychol..

[11] Finley A.J., Tang D., Schmeichel B.J. (2019). Sweet nothings: No effects of self-control exertion on blood glucose levels. Soc. Psychol..

[12] Inzlicht M., Schmeichel B.J. (2012). What Is Ego Depletion? Toward a Mechanistic Revision of the Resource Model of Self-Control. Perspect. Psychol. Sci..

[13] Inzlicht M., Schmeichel B.J., Macrae C.N. (2014). Why self-control seems (but may not be) limited. Trends Cogn. Sci..

[14] Baumeister R.F., Vohs K.D. (2016). Strength model of self-regulation as limited resource: Assessment, controversies, update. Adv. Exp. Soc. Psychol..

[15] Baumeister R.F., Tice D.M., Vohs K.D. (2018). The Strength Model of Self-Regulation: Conclusions From the Second Decade of Willpower Research. Perspect. Psychol. Sci..

[16] Baumeister R.F., Vohs K.D. (2007). Self-Regulation, Ego Depletion, and Motivation. Soc. Personal. Psychol. Compass.

[17] Sun H., Soh K.G., Norjali Wazir M.R.W., Ding C., Xu T., Zhang D. (2022). Can Self-Regulatory Strength Training Counter Prior Mental Exertion? A Systematic Review of Randomized Controlled Trials. Front. Public Health.

[18] Denson T.F., Capper M.M., Oaten M., Friese M., Schofield T.P. (2011). Self-control training decreases aggression in response to provocation in aggressive individuals. J. Res. Personal..

[19] MacMahon C., Schücker L., Hagemann N., Strauss B. (2014). Cognitive fatigue effects on physical performance during running. J. Sport Exerc. Psychol..

[20] Pires F.O., Silva-Júnior F.L., Brietzke C., Franco-Alvarenga P.E., Pinheiro F.A., de França N.M., Teixeira S., Santos T.M. (2018). Mental fatigue alters cortical activation and psychological responses, impairing performance in a distance-based cycling trial. Front. Physiol..

[21] Sun H., Soh K.G., Roslan S., Wazir M.R.W.N., Soh K.L. (2021). Does mental fatigue affect skilled performance in athletes? A systematic review. PLoS ONE.

[22] Sun H., Soh K.G., Xu X. (2022). Nature Scenes Counter Mental Fatigue-Induced Performance Decrements in Soccer. Front. Psychol..

[23] Soylu Y., Ramazanoglu F., Arslan E., Clemente F. (2022). Effects of mental fatigue on the psychophysiological responses, kinematic profiles, and technical performance in different small-sided soccer games. Biol. Sport.

[24] Cao S., Geok S.K., Roslan S., Sun H., Lam S.K. (2022). Mental Fatigue and Basketball Performance: A Systematic Review. Front. Psychol..

[25] Englert C. (2016). The strength model of self-control in sport and exercise psychology. Front. Psychol..

[26] Furley P., Bertrams A., Englert C., Delphia A. (2013). Ego depletion, attentional control, and decision making in sport. Psychol. Sport Exerc..

[27] McMorris T., Barwood M., Hale B.J., Dicks M., Corbett J. (2018). Cognitive fatigue effects on physical performance: A systematic review and meta-analysis. Physiol. Behav..

[28] Brown D.M.Y., Graham J.D., Innes K.I., Harris S., Flemington A., Bray S.R. (2020). Effects of Prior Cognitive Exertion on Physical Performance: A Systematic Review and Meta-Analysis.

[29] Proost M., Habay J., Wachter J.D., Pauw K.D., Rattray B. (2022). How to Tackle Mental Fatigue: A Systematic Review of Potential Countermeasures and Their Underlying Mechanisms. Sports Med..

[30] Hancock P.A., Desmond P.A. (2001). Stress, Workload and Fatigue.

[31] Boksem M.A.S., Tops M. (2008). Mental fatigue: Costs and benefits. Brain Res. Rev..

[32] Fortes L.S., De Lima-Junior D., Fiorese L., Nascimento-Júnior J.R.A., Mortatti A.L., Ferreira M.E.C. (2020). The effect of smartphones and playing video games on decision-making in soccer players: A crossover and randomised study. J. Sports Sci..

[33] Gantois P., Ferreira M.E.C., de Lima-Junior D., Nakamura F.Y., Batista G.R., Fonseca F.S., Fortes L.D.S. (2019). Effects of mental fatigue on passing decision- making performance in professional soccer athletes. Eur. J. Sport Sci..

[34] Englert C., Bertrams A., Furley P., Oudejans R.R.D. (2015). Is ego depletion associated with increased distractibility? Results from a basketball free throw task. Psychol. Sport Exerc..

[35] Pageaux B., Marcora S.M., Rozand V., Lepers R. (2015). Mental fatigue induced by prolonged self-regulation does not exacerbate central fatigue during subsequent whole-body endurance exercise. Front. Hum. Neurosci..

[36] Smith M.R., Marcora S.M., Coutts A.J. (2015). Mental fatigue impairs intermittent running performance. Med. Sci. Sports Exerc..

[37] Müller T., Apps M.A.J. (2019). Motivational fatigue: A neurocognitive framework for the impact of effortful exertion on subsequent motivation. Neuropsychologia.

[38] Pattyn N., Van Cutsem J., Dessy E., Mairesse O. (2018). Bridging exercise science, cognitive psychology, and medical practice: Is “cognitive fatigue” a remake of “the emperor’s new clothes”?. Front. Psychol..

[39] MacMahon C., Parrington L., Pickering T., Aitken B., Schücker L. Understanding the effects of cognitive tasks on physical performance: A constraints framework to guide further research. Int. Rev. Sport Exerc. Psychol..

[40] Smith M.R., Thompson C., Marcora S.M., Skorski S., Meyer T., Coutts A.J. (2018). Mental Fatigue and Soccer: Current Knowledge and Future Directions. Sports Med..

[41] Moher D., Shamseer L., Clarke M., Ghersi D., Liberati A., Petticrew M., Shekelle P., Stewart L., Prisma P. (2015). Preferred reporting items for systematic review and meta-analysis protocols (PRISMA-P) 2015 statement. Syst. Rev..

[42] Muraven M., Baumeister R.F., Tice D.M. (1999). Longitudinal Improvement of Self-Regulation Through Practice. J. Soc. Psychol..

[43] Nawrot P., Jordan S., Eastwood J., Rotstein J., Hugenholtz A., Feeley M. (2003). Effects of caffeine on human health. Food Addit. Contam..

[44] Dworzański W., Opielak G., Burdan F. (2009). Side effects of caffeine. Pol. Merkur Lek..

[45] Jacquet T., Poulin-Charronnat B., Bard P., Perra J., Lepers R. (2021). Physical Activity and Music to Counteract Mental Fatigue. Neuroscience.

[46] Kmet L.M., Lee R.C., Cook L.S. (2004). Standard Quality Assessment Criteria for Evaluating Primary Research Papers from a Variety of Fields.

[47] Wan E.W., Sternthal B. (2008). Regulating the Effects of Depletion Through Monitoring. Personal. Soc. Psychol. Bull..

[48] Allom V., Mullan B. (2015). Two inhibitory control training interventions designed to improve eating behaviour and determine mechanisms of change. Appetite.

[49] Emfield A.G., Neider M.B. (2014). Evaluating visual and auditory contributions to the cognitive restoration effect. Front. Psychol..

[50] Oaten M., Cheng K. (2006). Longitudinal gains in self-regulation from regular physical exercise. Br. J. Health Psychol..

[51] Bray S.R., Graham J.D., Saville P.D. (2015). Self-control training leads to enhanced cardiovascular exercise performance. J. Sports Sci..

[52] Filipas L., Martin K., Northey J.M., La Torre A., Keegan R., Rattray B. (2020). A 4-week endurance training program improves tolerance to mental exertion in untrained individuals. J. Sci. Med. Sport.

[53] Stocker E., Englert C., Seiler R. (2019). Self-control strength and mindfulness in physical exercise performance: Does a short mindfulness induction compensate for the detrimental ego depletion effect?. J. Appl. Sport Psychol..

[54] Shaabani F., Naderi A., Borella E., Calmeiro L. (2020). Does a brief mindfulness intervention counteract the detrimental effects of ego depletion in basketball free throw under pressure?. Sport Exerc. Perform. Psychol..

[55] Loch F., Hof zum Berge A., Ferrauti A., Meyer T., Pfeiffer M., Kellmann M. (2020). Acute effects of mental recovery strategies after a mentally fatiguing task. Front. Psychol..

[56] Graham J.D., Bray S.R., Martin Ginis K.A. (2014). “Pay the piper”: It helps initially, but motivation takes a toll on self-control. Psychol. Sport Exerc..

[57] Brown D.M.Y., Bray S.R. (2019). Heart rate biofeedback attenuates effects of mental fatigue on exercise performance. Psychol. Sport Exerc..

[58] Webb T.L., Sheeran P. (2003). Can implementation intentions help to overcome ego-depletion?. J. Exp. Soc. Psychol..

[59] Oaten M., Cheng K. (2006). Improved self-control: The benefits of a regular program of academic study. Ter. Arkhiv.

[60] Gailliot M.T., Plant E.A., Butz D.A., Baumeister R.F. (2007). Increasing self-regulatory strength can reduce the depleting effect of suppressing stereotypes. Personal. Soc. Psychol. Bull..

[61] Oaten M., Cheng K. (2007). Improvements in self-control from financial monitoring. J. Econ. Psychol..

[62] Bertrams A., Schmeichel B.J. (2014). Improving self-control by practicing logical reasoning. Self Identity.

[63] Miles E., Sheeran P., Baird H., Macdonald I., Webb T.L., Harris P.R. (2016). Does self-control improve with practice? Evidence from a six-week training program. J. Exp. Psychol. Gen..

[64] Friese M., Messner C., Schaffner Y. (2012). Mindfulness meditation counteracts self-control depletion. Conscious. Cogn..

[65] Axelsen J.L., Kirk U., Staiano W. (2020). On-the-spot binaural beats and mindfulness reduces the effect of mental fatigue. J. Cogn. Enhanc..

[66] Laumann K., Gärling T., Stormak K.M. (2003). Selective attention and heart rate responses to natural and urban environments. J. Environ. Psychol..

[67] Berto R. (2005). Exposure to restorative environments helps restore attentional capacity. J. Environ. Psychol..

[68] Berman M.G., Jonides J., Kaplan S. (2008). The cognitive benefits of interacting with nature. Psychol. Sci..

[69] Beute F., de Kort Y.A.W. (2014). Natural resistance: Exposure to nature and self-regulation, mood, and physiology after ego-depletion. J. Environ. Psychol..

[70] Chow J.T., Lau S. (2015). Nature gives us strength: Exposure to nature counteracts ego-depletion. J. Soc. Psychol..

[71] Lee K.E., Williams K.J.H., Sargent L.D., Williams N.S.G., Johnson K.A. (2015). 40-second green roof views sustain attention: The role of micro-breaks in attention restoration. J. Environ. Psychol..

[72] Evensen K.H., Raanaas R.K., Hagerhall C.M., Johansson M., Patil G.G. (2015). Restorative elements at the computer workstation: A comparison of live plants and inanimate objects with and without window view. Environ. Behav..

[73] Pilotti M., Klein E., Golem D., Piepenbrink E., Kaplan K. (2015). Is viewing a nature video after work restorative? Effects on blood pressure, task performance, and long-term memory. Environ. Behav..

[74] Haga A., Halin N., Holmgren M., Sörqvist P. (2016). Psychological restoration can depend on stimulus-source attribution: A challenge for the evolutionary account?. Front. Psychol..

[75] Zhang Y., Kang J., Kang J. (2017). Effects of Soundscape on the Environmental Restoration in Urban Natural Environments. Noise Health.

[76] Bennett M. (2019). The Effect of Sound on Attention Restoration.

[77] Neilson B.N., Craig C.M., Curiel R.Y., Klein M.I. (2021). Restoring attentional resources with nature: A replication study of Berto’s (2005) paradigm Including commentary from Dr. Rita Berto. Hum. Factors.

[78] Tyler J.M., Burns K.C. (2008). After depletion: The replenishment of the self’s regulatory resources. Self Identity.

[79] Boucher H.C., Kofos M.N. (2012). The idea of money counteracts ego depletion effects. J. Exp. Soc. Psychol..

[80] Zhu Z., Li J., Zhang B., Li Y., Zhang H. (2017). The effect of motivation and positive affect on ego depletion: Replenishment versus release mechanism. Int. J. Psychol..

[81] Brown D.M.Y., Bray S.R. (2017). Effects of mental fatigue on physical endurance performance and muscle activation are attenuated by monetary incentives. J. Sport Exerc. Psychol..

[82] Muraven M., Slessareva E. (2003). Mechanisms of self-control failure: Motivation and limited resources. Society.

[83] Gollwitzer P.M. (1999). Implementation intentions: Strong effects of simple plans. Am. Psychol..

[84] Gollwitzer P.M., Schaal B. (1998). Metacognition in action: The importance of implementation intentions. Personal. Soc. Psychol. Rev..

[85] Brown D.M.Y., Bray S.R. (2019). Effects of Mental Fatigue on Exercise Intentions and Behavior. Ann. Behav. Med..

[86] Baumeister R.F., Vohs K.D. (2004). Handbook of Self-Regulation.

[87] Berto R., Baroni M.R., Zainaghi A., Bettella S. (2010). An exploratory study of the effect of high and low fascination environments on attentional fatigue. J. Environ. Psychol..

[88] Smith M.R., Fransen J., Deprez D., Lenoir M., Coutts A.J. (2017). Impact of mental fatigue on speed and accuracy components of soccer-specific skills. Sci. Med. Footb..

[89] Xiao S., Dang J., Mao L., Liljedahl S. (2014). When more depletion offsets the ego depletion effect. Soc. Psychol..

[90] Baumeister R.F. (2002). Ego depletion and self-control failure: An energy model of the self’s executive function. Self Identity.

[91] Kamitsis I., Francis A.J.P. (2013). Spirituality mediates the relationship between engagement with nature and psychological wellbeing. J. Environ. Psychol..

[92] Chen Z., Gan K.K., Zhou T., Du Q., Zeng M. (2022). Using Structural Equation Modeling to Examine Pathways Between Environmental Characteristics and Perceived Restorativeness on Public Rooftop Gardens in China. Front. Public Health.

[93] Malekinezhad F., Courtney P., Bin Lamit H., Vigani M. (2020). Investigating the Mental Health Impacts of University Campus Green Space Through Perceived Sensory Dimensions and the Mediation Effects of Perceived Restorativeness on Restoration Experience. Front. Public Health.

[94] Kaplan R., Kaplan S. (1898). The Experience of Nature: A Psychological Perspective.

[95] Spitzer U.S., Hollmann W. (2013). Experimental observations of the effects of physical exercise on attention, academic and prosocial performance in school settings. Trends Neurosci. Educ..

[96] Zang Y. (2019). Impact of physical exercise on children with attention deficit hyperactivity disorders: Evidence through a meta-analysis. Medicine.

[97] Posner M.I., Snyder C.R., Solso R. (2004). Attention and cognitive control. Cogn. Psychol. Key Read..

[98] Kaplan S., Berman M.G. (2010). Directed attention as a common resource for executive functioning and Self-Regulation. Perspect. Psychol. Sci..

[99] Inzlicht M., Werner K.M., Briskin J.L., Roberts B.W. (2021). Integrating Models of Self-Regulation. Annu Rev. Psychol..

[100] Carver C.S., Scheier M.F. (1982). Control theory: A useful conceptual framework for personality-social, clinical, and health psychology. Psychol. Bull..

[101] Fortes L.S., Gantois P., de Lima-Júnior D., Barbosa B.T., Ferreira M.E.C., Nakamura F.Y., Albuquerque M.R., Fonseca F.S. (2021). Playing videogames or using social media applications on smartphones causes mental fatigue and impairs decision-making performance in amateur boxers. Appl. Neuropsychol. Adult.

[102] Filipas L., Ferioli D., Banfi G., La Torre A., Vitale J.A. (2021). Single and Combined Effect of Acute Sleep Restriction and Mental Fatigue on Basketball Free-Throw Performance. Int. J. Sports Physiol. Perform..

[103] Van Cutsem J., De Pauw K., Vandervaeren C., Marcora S., Meeusen R., Roelands B. (2019). Mental fatigue impairs visuomotor response time in badminton players and controls. Psychol. Sport Exerc..

[104] Fortes L.S., Lima-Junior D.D., Gantois P., Nasicmento-Junior J.R.A., Fonseca F.S. (2021). Smartphone Use Among High Level Swimmers Is Associated With Mental Fatigue and Slower 100-and 200-but Not 50-Meter Freestyle Racing. Percept. Mot. Ski..

[105] Weerakkody N.S., Taylor C.J., Bulmer C.L., Hamilton D.B., Gloury J., O’Brien N.J., Saunders J.H., Harvey S., Patterson T.A. (2021). The effect of mental fatigue on the performance of Australian football specific skills amongst amateur athletes. J. Sci. Med. Sport.

[106] Martin K., Thompson K.G., Keegan R., Rattray B. (2019). Are Individuals Who Engage in More Frequent Self-Regulation Less Susceptible to Mental Fatigue?. J. Sport Exerc. Psychol..

