# Supplementary material for: The Counteractive Effect of Self-Regulation-Based Interventions on Prior Mental Exertion: A Systematic Review of Randomised Controlled Trials
Source: Brain Sci. 2022 Jul 8;12(7):896. doi: 10.3390/brainsci12070896 (PMC9313235; doi:10.3390/brainsci12070896)
Supplement: Supplementary file 1 [file brainsci-12-00896-s001.zip › brainsci-1725691-supplementary.pdf]

Table S1 - Detailed search strategy

Database	Search Keywords	Results
Pubmed	(1) (((“mental fatigue”) OR (“mental exertion”)) OR (“cognitive fatigue”)) OR (“ego depletion”), 3290<br>(2) ((((((((intervention) OR (treatment)) OR (restor*)) OR (counter*)) OR (attenuate)) OR (improv*)) OR (protect*)) OR (overcome)) OR (“self-regulatory strategy”), 18, 595, 122<br>(3) (1) AND (2), 2074<br>(4) (3) limits published date 1999 - Feb 2022, 1836<br>(5) (4) limits randomized control trial, 242	242
Web of Science	(1) TS=("mental fatigue" OR "mental exertion" OR "cognitive fatigue" OR "ego depletion"), 6162<br>(2) TS=(intervention OR treatment OR restor* OR counter* OR attenuate OR improv* OR protect* OR overcome OR “self-regulatory strategy”), 17,558,479<br>(3) (1) AND (2), 2174<br>(4) (3) limits published date: 1999 – Feb 2022, 2068	1946

	(5) (4) limits English, 1946	
EBSCOhost	(1) "mental fatigue" OR "mental exertion" OR "cognitive fatigue" OR "ego depletion" AND intervention OR treatment OR restor* OR counter* OR attenuate OR improv* OR protect* OR overcome OR “self-regulatory strategy”, 823<br>(2) (1) limits published date: 1999 – Feb 2022, 815<br>(3) (2) limits “Academic Journal”, 810	810
Scopus	(1) TITLE-ABS-KEY(("mental fatigue" OR "mental exertion" OR "cognitive fatigue" OR "ego depletion") AND (intervention OR treatment OR restor* OR counter* OR attenuate OR improv* OR protect* OR overcome OR “self-regulatory strategy”)), 1648<br>(2) (1) limited English, 1512<br>(3) (2) limited to 1999, 1451	1451

Noted. * the symbol of the truncation wildcard.

Table S2: "Qualsyst" of quality assessment

Publication	Question/ objective described	Appropriate study design	Appropriate subject selection	Characteristics sufficiently described	Random allocation	Researchers blinded	Subjects blinded	Outcome measures well defined and robust to bias	Appropriate sample size	Analytic methods well described	Estimate of variance reported	Controlled for confounding	Results reported in detail	Conclusion supported by results?	Rating
Webb and Sheeran (2003)	2	2	1	1	2	0	0	2	1	2	2	0	2	2	Medium
Echo Wen and Sternthal (2008)	2	2	1	0	0	0	0	2	1	2	2	2	2	2	Medium
Brown and Bray (2019)	2	2	1	2	2	0	2	2	2	2	2	2	1	2	High
Muraven et al. (1999)	2	2	1	0	2	0	2	1	0	1	1	0	2	2	Medium
Oaten and Cheng (2006a)	2	2	1	0	2	0	0	2	0	2	1	0	2	2	Medium
Oaten and Cheng (2006b)	2	2	1	0	2	0	0	2	0	2	1	0	2	2	Medium
Gailliot et al. (2007)	2	2	1	0	2	0	0	2	1	1	1	0	2	2	Medium
Oaten and Cheng (2007)	2	2	1	1	2	1	0	2	1	1	1	1	2	2	Medium
Denson et al. (2011)	2	2	0	2	2	0	0	2	1	1	1	0	2	2	Medium
Cranwell et al.	1	2	1	1	2	0	0	2	1	1	2	0	2	1	Medium

(2014)															
Bertrams and Schmeichel (2014)	2	2	1	1	2	0	0	2	1	1	1	0	2	2	Medium
Bray et al. (2015)	2	2	1	2	2	0	0	2	2	2	2	2	2	2	High
Allom and Mullan (2015)	2	2	1	2	1	0	0	1	2	2	1	0	2	2	Medium
Miles et al. (2016)	2	2	1	0	2	0	0	2	2	0	1	0	2	2	Medium
Filipas et al. (2020)	2	2	0	0	2	2	2	2	1	2	1	0	2	2	Medium
Friese et al. (2012)	2	2	0	1	2	0	0	2	1	0	2	0	2	2	Medium
Stocker et al. (2019)	2	2	1	1	1	2	0	1	2	0	2	0	2	2	Medium
Axelsen et al. (2020)	2	2	2	1	0	0	2	2	1	2	1	0	2	2	Medium
Shaabani et al. (2020)	2	2	2	1	2	2	0	1	1	2	1	0	2	2	Medium
Laumann et al. (2003)	2	2	0	1	2	0	0	2	1	2	1	0	2	2	Medium
Berto (2005)	2	2	1	1	2	0	0	2	1	0	1	0	2	2	Medium
Berman et al. (2008)	2	2	0	1	2	0	0	2	0	1	1	1	2	2	Medium
Valtchanov et al. (2010)	1	2	1	0	2	0	0	1	0	1	1	0	1	1	Low
Beute and de Kort	2	2	1	1	0	0	0	2	1	2	2	2	2	2	Medium

(2014)														
Emfield and Neider (2014)	2	2	1	1	2	0	0	2	2	2	2	2	2	2	High
Chow and Lau (2015)	1	2	1	1	2	0	0	1	1	0	1	2	2	2	Medium
Lee et al. (2015)	2	2	1	1	2	0	0	2	1	2	2	0	2	2	Medium
Lobo et al. (2015)	1	2	1	1	2	0	0	1	1	1	2	0	2	1	Low
Evensen et al. (2015)	2	2	1	1	2	0	0	2	1	2	2	0	2	2	Medium
Dang et al. (2015)	1	2	1	1	2	0	0	1	1	1	1	1	2	1	Low
Pilotti et al. (2015)	2	2	0	1	2	0	0	2	1	2	1	0	2	2	Medium
Haga et al. (2016)	2	2	1	1	2	0	0	2	1	0	1	0	2	2	Medium
Zhang et al. (2017)	2	2	2	1	0	0	0	2	1	2	2	1	2	2	Medium
Bennett (2019)	2	2	1	1	0	0	0	2	1	1	2	0	2	2	Medium
Neilson et al. (2020)	2	2	1	1	2	0	0	2	1	0	1	0	2	2	Medium
Tyler and Burns (2008)	2	2	0	1	2	0	0	2	1	0	2	1	2	2	Medium
Loch et al. (2020)	2	2	1	1	2	0	0	2	2	2	1	0	2	2	Medium
Muraven and Slessareva (2003)	2	2	1	1	0	0	0	2	1	1	1	1	2	2	Medium
Boucher and Kofos (2012)	2	2	1	2	2	0	0	2	1	1	1	2	2	2	Medium

Graham et al. (2014)	2	2	1	1	2	0	2	2	1	1	2	2	2	2	High
Zhu et al. (2017)	2	2	2	1	2	0	0	1	1	1	1	0	2	2	Medium
Brown and Bray (2017)	1	2	1	1	2	0	2	2	2	2	1	0	2	2	Medium

Table S3: Characteristics of standard manipulated interventions details

NO	Publication	Mental Exertion	Duration	Subject (N)	Intervention Description and Specific Method	Intervention Based Theory	Outcome	Context of the Study
1	Webb and Sheeran (2003)<br>study 2	Mathematical test	UA	57 Undergraduates	Implementation intention: specified cues and achieve strategic automatization of SR.<br>“As long as I see the word, I will ignore its meaning and I will name the color ink it is printed in.”	The resource model of self-regulation	C: Stroop task<br>Completion time↓<br>Error Rate↓	Healthy behavior

P: physical task; C: cognitive task; UA: unavailable; SR: self-regulation; M: matched.

Table S4: Characteristics of monitoring manipulated interventions details

NO	Publication	Mental Exertion	Duration	Subject (N)	Intervention Description and Specific Method	Intervention Based Theory	Outcome	Context of the Study
1	Echo Wen and Sternthal (2008)	Cross-off-the-letter task	10 min	study 1: 28 (28♀)<br>study 2: 81 (47♀)<br>study 4: 83 (48♀)<br>Undergraduates	Monitoring time: monitoring entails comparing subjects' behavior to a salient performance standard with the goal of adjusting the behavior to minimize the discrepancy between current performance and the standard.<br>Provide accurate moment-to-moment feedback about the time they had spent on the puzzle task.	The resource model of self-regulation	C: Puzzle task: Persistence time↑	Health behavior
2	Brown and Bray (2019)	Stroop task	10 min	36<br>Sex: 16♂; 20♀<br>Age: 19.44 ± 1.42<br>University Students	HR biofeedback: self-regulation of behavior is enabled through a process of setting a goal and monitoring behavior using feedback to adjust goal-behavior discrepancies and facilitate goal attainment.<br>Provide biofeedback about HR on the cycle ergometer.	Control theory	P: Total exercise load: Accumulated energy↑	Exercise

P: physical task; C: cognitive task; UA: unavailable; SR: self-regulation; SST: scrambled sentence task HR: heart rate; M: matched; ♀: female; ♂: male.

Table S5: Characteristics of strength manipulated interventions (preventing before mental exertion) details

NO	Publication	Mental Exertion	Duration	Subject (N)	Intervention Description and Specific Method	Intervention Based Theory	Outcome	Similarity	Context of the Study
1	Muraven et al. (1999)	Thought suppression task	5 min	69<br>Sex: 42♂; 27♀<br>Undergraduates	*Repeated exercise:* increase self-regulatory strength like a muscle may counter mental exertion.<br>**2 weeks** posture adjustment.	The resource model of self-regulation	P: Handgrip:<br>Persistence time↑	M	Social behavior
2	Oaten and Cheng (2006a)	Thought suppression task	5 min	45<br>Sex: 7♂; 38♀<br>Undergraduates	*Regular exercise:* (i) increase awareness to these suggested versus enacted discrepancies (ii) learn to modify behaviors and enhance SR<br>**8 weeks** work on the academic program and achieve commitments.	The resource model of self-regulation	C: Inhibition<br>Visual tracking task:<br>Error↓ in the exam period<br>Self-efficacy ↔<br>Perceived Stress ↔<br>Emotional distress ↔	M	Academic performance
3	Oaten and Cheng (2006b)	Thought suppression task	5 min	24<br>Sex: 6♂; 18♀<br>Age: 24±6<br>Undergraduates	*Repeated exercise:* Regular physical exercise based on the Muraven et al. (1999).<br>**2 months** of aerobic classes, free-weights, and resistance training.	The resource model of self-regulation	C: Inhibition<br>Visual track task:<br>Error rate↓	U	Exercise
4	Gailliot et al. (2007)<br>Study 1	Stereotype-suppression task	UA	40<br>Sex: 24♂; 14♀<br>Undergraduates	*Repeated exercise:* change habit of speaking and gain SR.<br>**2 weeks** modifying verbal mannerism: e.g., only say “yes” and “no” instead of using similar colloquialisms and avoid using	The resource model of self-regulation	C: Problem-solving<br>Anagram task:<br>Number↑ in low-motivation group;<br>Number ↔ in high-motivation group	U	Social behavior

					sentences that began with “I”.				
	Study 2	Stereotype-suppression task	UA	98<br>Sex: 31♂; 67♀<br>Undergraduates	*Repeated exercise:* increase self-regulatory strength like a muscle may counter mental exertion.<br>**2 weeks** non-dominant hand using (e.g., brushing their teeth, opening doors, computer mouse using)		C: Problem-solving<br>Anagram task:<br>Number↑ in low-motivation group;<br>Number ↔ in high-motivation group<br><br>Low and high motivation group Mood ↔<br>Arousal ↔	U	
	Study 4	Stereotype-suppression task	UA	53<br>Sex: 11♂; 41♀<br>Undergraduates	*Repeated exercise:* increase self-regulatory strength like a muscle may counter mental exertion.<br>**2 weeks** *non dominant hand using (same as study 2)*		C: Inhibition<br>Stroop task:<br>Accuracy↑ in low motivation group<br>Accuracy ↔ in high-motivation group	M	
5	Oaten and Cheng (2007)	Thought-suppression task	5 min	60<br>Sex: 12♂; 37♀<br>Undergraduates	*Repeated exercise:* increase self-regulatory strength like a muscle may counter mental exertion.<br>**4 months** financial monitoring program to gain SR	The resource model of self-regulation	C: Inhibition<br>Visual tracking task:<br>Error↓<br>Self-efficacy ↔<br>Perceived stress ↔<br>Emotional distress ↔	M	Social behavior
6	Denson et al. (2011)	Anger induction	12 min	70<br>Sex: 16♂; 54♀<br>Age: 20.30±2.99<br>Undergraduates	*Repeated exercise:* increase self-regulatory strength like a muscle may counter mental exertion.<br>**2 weeks** non-dominant hand using	The resource model of self-regulation	C: Inhibition<br>Visual tracking task:<br>Error↓<br>Self-efficacy ↔<br>Perceived stress ↔<br>Emotional distress ↔	M	Social behavior

7	Cranwell et al. (2014)<br>Study 1	Stroop task	10 min	29<br>Sex: 29♀<br>University students and staff	*Repeated exercise:* Stroop-task Internet-based application via smartphone. Transfer previous results to smartphone application.<br>7 days per week for **4 weeks** practice on the smartphone.	The resource model of self-regulation	C: Inhibition<br>Stroop task:<br>Response latency scores↑<br>P: Handgrip<br>Persistence time↑	M	Social behavior
	Study 2	Complex counting task	UA	33<br>Sex: 33♀<br>University students and staff				U	
8	Bertrams and Schmeichel (2014)	Typing task	UA	49<br>Sex: 11♂; 38♀<br>Age: 22.49±3.50<br>Undergraduates	*Repeated exercise (logical reasoning practice):* improve executive capacity to become more resistant to depletion.<br>**1 week** of daily regular practice at logical reasoning. Each day subjects write down arguments about 10 controversial statements.	The resource model of self-regulation	C: Problem-solving<br>Anagram task:<br>Numbers of solved anagrams↑	M	Social behavior
9	Bray et al. (2015)	Stroop task	5 min	41<br>Sex: 15♂; 26♀<br>Age: 18.66±1.56<br>Undergraduates	*Repeated exercise (logical reasoning practice):* improve executive capacity to become more resistant to depletion.<br>**2 weeks** isometric handgrip exercise (squeeze the handles tightly together for as long as possible, twice daily with dominant hand.	The resource model of self-regulation	P: Maximal cardiovascular exercise:<br>Time to fail↑<br>RPE ↔	U	Exercise
10	Allom and Mullan (2015)<br>Study 1	Letter typing task	5 min	82<br>Sex: 16♂; 66♀<br>Age: 20.43±4.86<br>Undergraduates	*Repeated exercise* (logical reasoning practice): improve executive capacity to become more resistant to depletion.<br>**10 days** food specific inhibition: the stop-signal was only presented after unhealthy food images.<br>General inhibition: the stop-signal was randomly presented either after a healthy or an unhealthy food image	The resource model of self-regulation	C: Inhibition<br>Vulnerability to depletion↓<br>20 number trails of Stroop task:<br>Exp 1 vs. Con:<br>Reaction time ↔<br>Accuracy ↔<br>Exp 2 vs. Con<br>Reaction time ↔<br>Accuracy ↔	M	Heath behavior
	Study 2	Letter typing task	5 min	78<br>University students and staff	Same as study 1		C: Inhibition<br>Vulnerability to depletion↓	M	

							50 number trails of Stroop task:<br>Exp 1 vs. Con:<br>Reaction time↓<br>Exp 2 vs. Con<br>Reaction time↓<br>Follow-up test ↔		
11	Miles et al. (2016)	Four consecutive tasks that	UA	174<br>Sex: 71♂; 103♀<br>undergraduate and postgraduate students	*Repeated exercise* (regular behavioral and cognitive training program): Increase self-regulatory strength like a muscle may counter mental exertion.<br>Behavioral training intervention: using one's non-dominant hand for all daily activities.<br>Cognitive training intervention: perform the Stroop task and the stop-signal task (both interventions are 5 days per week in total **6 weeks**).	The resource model of self-regulation	P: Handgrip<br>Persistence time ↔	M	Social behavior
12	Filipas et al. (2020)	45-min cognitive battery, 40-min Stroop task and 5-min flanker ask	90 min	20<br>Sex: 6♂; 14♀<br>Age: 27.6±6.2<br>Untrained individuals	*Repeated exercise* (endurance training program): endurance exercise is linked to cognitive benefits; brain adaptations to physical training could therefore also be important in our resistance to mental fatigue<br>**4 weeks** of the incremental maximal ramp test. Each week training consisted of 1×60 min at 65-70%; 1×20 min at 65-70%, plus 6×3 min at 85-90%; 1×20 min at 65-70% followed by 40 min at 75-80% of peak HR	UA	P: Cycling ergometer<br>Total distance↑	M	Exercise

P: physical task; C: cognitive task; SR: self-regulation; UA: unavailable; BET: brain endurance training; HR: heart rate; M: matched; U: unmatched; ♀: female; ♂: male.

Table S6: Characteristics of strength manipulated interventions (recover after mental exertion) details

NO	Publication	Mental Exertion	Duration	Subject (N)	Intervention Description and Specific Method	Intervention Based Theory	Outcome	Similarity	Context of the Study
**Mindfulness Meditation**									
1	Friese et al. (2012)	Emotion suppression task	5 min	66<br>Sex: 26♂; 40♀<br>Age: 43.27±11.91	*Mindfulness meditation*: improve attention regulation and awareness.<br>A **brief** meditation. Noted: at the end of a meditation seminar, subjects were approached.	The resource model of self-regulation	C: Attention<br>d2 Test of attention<br>Correct numbers↑	M	Social behavior
2	Stocker et al. (2019)	Transcription task	5 min	34<br>Sex: 16♂; 18♀<br>Age: 20.85±1.31<br>Sport students	*Mindfulness meditation:* improve attention regulation and/or executive function<br>**4-min** mindfulness exercise via audio.	The resource model of self-regulation	P: Pland exercise<br>Persistence time ↔	U	Exercise
3	Axelsen et al. (2020)	AX-continuous performance test	90 min	90<br>Sex: 43♂; 47♀	*Mindfulness meditation:* enhanced cognitive performance including increased sustained attention and working memory<br>Listen to a guided mindfulness audio file **12min** that subjects listened to with headphones.	UA	C: Attention<br>SART %NoGo<br>Success Rate (EMG)↑<br>Success Rate (NG)↓	M	Working behavior

4	Shaabani et al. (2020)	Stroop task	15 min	72<br>Sex: 72♂<br>Age: 28.6±4.0<br>Basketball players	*Mindfulness meditation:* reduce the overload of cognitive resources.<br>The mindfulness intervention consisted of a **15-min** audio for: 1) ‘‘focused breathing induction’’, where the participants focused on their breathing; 2) “breathing and body mindfulness”.	The resource model of self-regulation	P: Basketball free throw<br>Shooting score↑	U	Sport performance
	**Nature Exposure**								
5	Laumann et al. (2003)	Proofreading task	15 min	28<br>Sex: 28♀<br>Age: 14 – 18 yr<br>Undergraduates	*Nature exposure: n*ature surrounding restore depleted voluntary attention.<br>**20min** nature scenes video, depicting waterside environment.	ART	C: Attention<br>Posner’s attention-orienting task<br>Valid trials for the exogenous↑	M	Academic performance
6	Berto (2005)<br>Study 1	SART	5 min	32<br>Age: 23±3.22<br>Undergraduates	*Nature exposure:* restorative environments facilitate recovery from mental<br>fatigue.<br>25 restorative pictures, each picture was showed 15s on the computer. **6.25min**	ART	C: Attention<br>SART<br>D-prime; Reaction time and correction responses↑	M	Social behavior
	study 2				25 geometrical patterns, each pattern was showed 15s on the computer. **6.25min**		D-prime and Reaction time incorrect responses↑		
	study 3				25 restorative pictures, self-space exposure time, which was less than 15s for each picture. **Less than 6.25min**		D-prime and correction responses↑		
7	Berman et al. (2008)<br>study 1	Directed-forgetting task	35 min	38<br>Sex: 15♂; 23♀<br>Age: mean = 22.62<br>University students	*Nature exposure:* interaction with nature restore directed attention.<br>**50-55min** walk in nature	ART	C: Working memory<br>Backwards digit-span Corrections↑	U	Social behavior

	study 2			12<br>Sex: 4♂; 8♀<br>Age: mean = 24.25<br>University students	50 nature pictures were showed 7s of each. **5.83min**		C: Working memory<br>Backwards digit-span<br>Corrections↑<br>Attention network test:<br>Executive functions↑<br>alerting and orienting↓	U	
8	Beute and de Kort (2014)<br>Study 1	Typing task	UA	90<br>Sex: 42♂; 48♀<br>Age: 22.2±3.1	*Nature exposure:* improve self-regulation and executive function.<br>Subjects rated the presented slideshow about nature**. 3min**: 20s each, 9 pictures.	(i) ART<br>(ii) The resource model of self-regulation	C: Inhibition<br>Stroop task<br>Reaction time↓<br>Errors ↔	U	Social behavior
	Study 2	Typing task and Stroop task	Stroop task 4 min	121<br>Sex: 70♂; 51♀<br>Age: 21.1±2.2			C: 2-back task<br>Reaction time ↔<br>Errors ↔		
9	Emfield and Neider (2014)	Cognitive battery task (3 types)	30 min	202<br>Sex: 74♂; 128♀<br>Age: mean = 19.8<br>Undergraduates	*Nature exposure:* nature scenes restore directed attention.<br>**5.83min**: 50 nature photos, 7s each; nature sound exposure; nature pictures + sound.	ART	C: Working memory<br>Backward digit span<br>Remember words ↔<br>Attention network task<br>Corrections ↔<br>FFOV<br>Accuracy↔	U	Health behavior
10	Chow and Lau (2015)<br>Study 1	Cross-off-the-letter task	10 min	42<br>Sex: 14♂; 28♀<br>Age: 20.81±2.46<br>Undergraduates	*Nature exposure:* (1) the self-regulatory strength can be replenished via rest; (2) rich in soft fascination helps people recover from mental fatigue and improve directed attention.<br>Subjects were provided a picture-album that consisted of natural settings for **6min.**	(i) ART<br>(ii) The resource model of self-regulation	C: Problem-solving<br>Anagram task<br>Persistence time↑	U	Academic performance
	Study 2	Perception task	6 min	58<br>Sex: 22♂; 36♀<br>Age: 19.79±0.99<br>Undergraduates	Subjects viewed nature scenes of slides. **4min:** 4 pictures, each 1min.		C: Reasoning<br>Logical reasoning task<br>Logical scores↑	U	

	Study 3	Retyping task	6 min	185<br>Sex: 63♂; 122♀<br>Age: 38.58±11.94	Subjects were asked to view 9 nature pictures and clicked on the area of the pictures that attracted their attention. **1.5min:** 9 picture, 10s each.		C: Problem-solving<br>Anagram task<br>Number of solved anagrams↑	U	
11	Lee et al. (2015)	SART	5 min	150<br>Sex: 43♂; 107♀<br>Age: mean = 20<br>University students	*Nature exposure: n*ature in cities is restorative.<br>Subjects self-determining length of viewing time; viewed a "green roof" planted with a meadow containing taller green grass and yellow flower. **40s**	(i) ART<br>(ii) Attention-resource model	C: Attention<br>SART<br>Omission, errors, slow-frequency gradual response and fast-frequency moment-to moment response↓	M	Working performance
12	Evensen et al. (2015)	Computer work	1h	85<br>Sex: 28♂; 57♀<br>Age: 24.9±5.7<br>Students	*Nature exposure:* Nature renew psychological resources that have been depleted; positive affect.<br>Four plants as the interior; inanimate objects replicated plants. **1h work with 5min break.**	(i) ART<br>(ii) Stress Recovery Theory	C: Working memory<br>Reading span task<br>Correct words↑	U	Working performance
13	Pilotti et al. (2015)	A workday	1day	63<br>Sex: 24♂; 39♀<br>Age: 31.79±9.42<br>Student advisors	*Nature exposure: n*ature scenes are good at physiological indices, performance, and self-report measures of well-being.<br>**15min** video about nature.	ART	C: Attention<br>Sustained attention task<br>Response latencies↔<br>Memory test<br>Long-term memory↑	M	Working performance
14	Haga et al. (2016)	A cognitive demanding task	40min	90<br>Sex: 29♂; 61♀<br>Age: 24.76±4.60<br>University students	*Nature exposure:* natural settings have greater restorative effects on psychological resources recovery.<br>**3min** with sound about nature environment with a streaming waterfall.	ART	C: Attention<br>Attention network test<br>Response-time ↔<br>Accuracy ↔	M	Health behavior
15	Zhang et al. (2017)	Reasoning test	50min	72<br>Sex: 36♂; 36♀<br>University students	*Nature exposure*: nature promote the restoration of people from the state of direction attention fatigue.	ART	C: Attention<br>Complement test<br>Grade of	M	Social behavior

					**40min** nature sound (birds, water, etc.) in the real environment.		complementary pairs↑		
16	Bennett (2019)	Math problems task	15min	116<br>Sex: 27♂; 89♀<br>Age: 18-51<br>University students	*Nature exposure:* nature settings attract involuntary attention, restoring directed attention.<br>Expose nature sound with headphones (bird songs). **4min**	ART	C: Working memory<br>Backwards digit-span<br>Corrections ↔	U	Social behavior
17	Neilson et al. (2020)	SART	5min	60<br>Sex: 30♂; 30♀<br>Age: 19.62±1.98	*Nature exposure:* restorative environments facilitate recovery from mental fatigue.<br>25 restorative pictures, each picture was showed 15s on the computer. **6.25min**	ART	C: Attention<br>SART<br>Reaction time and correction responses ↔	M	Social behavior
	**Other Recovery Strategy**								
18	Tyler and Burns (2008)<br>Study 1	A physical activity while performing a numerical exercise.	6min	60<br>Sex: 14♂; 46♀<br>undergraduate psychology students	*Relaxation:* it may be a crucial element in the effective replenishment of the self's depleted resource, as the sleep.<br>**1, 3 and 10min** interval between depletion and handgrip task.	The resource model of self-regulation	P: Inhibition<br>Handgrip task<br>Persistence time<br>1min↓<br>3min↓<br>10min↑	U	Social behavior
	Study 2	Thought-listing task	6min	40<br>Sex: 13♂; 27♀<br>undergraduate psychology students	**3min** relaxing interval about listening to a CD				
19	Loch et al. (2020)	AX-continuous performance test	60min	24<br>Age: 22.8±3.6<br>undergraduate and graduate students	*Mental recovery*: obtain baseline levels of mental abilities and the restoration of mental energy.<br>(i) Powernap: 20-min nap on a bed in a comfortable lying position.<br>(ii) Systematic breathing: A breathing rhythm twice as long as the inhalation period.	(i) The resource model of self-regulation<br>(ii) Psychobiological model	C: Problem-solving<br>math multiplication problems<br>Persistence time↑<br>C: Reaction time test<br>Reaction time↑	U	Sport performance

					(iii) Systematic breathing plus mental imagery systematic breathing intervention were adopted and linked to an imaginative period in which the participants visualized a self-selected relaxing picture.				

P: physical task; C: cognitive task; UA: unavailable; SART: sustained attention response Task; EMG: experienced mindfulness group; NG: novice group. ART: attention restoration theory. M: matched; U: unmatched; ♀: female; ♂: male.

Table S7: Characteristics of motivation manipulated interventions details

NO	Publication	Mental Exertion	Duration	Subject (N)	Intervention Description and Specific Method	Intervention Based Theory	Outcome	Context of the Study
1	Muraven and Slessareva (2003)<br>Study 2	Speech control	30min	82<br>Sex: 13♂; 27♀<br>Undergraduates	*Motivation:* diminishes when the chance of success seems remote.<br>Subjects were warned that the game is very difficult, and practice has little effect on final performance.	The resource model of self-regulation	C: A frustrating task<br>Persistence time↓	Social behavior
2	Boucher and Kofos (2012)<br>Experiment 1	Cross-off-the-letter task	5min	63<br>Sex: 17♂; 46♀<br>Age: mean = 18.6<br>College students	*Motivation:* actual money can buffer ego depletion.<br>Idea money: subjects were given five words and asked to use four of the words to create a meaningful sentence about money. Such as “won green the lottery I” became “I won the lottery”	The resource model of self-regulation	C: Cognitive task<br>Performance scores↑	Social behavior
	Experiment 2	Thought listing task	5min	44<br>Sex: 12♂; 22♀<br>Age: mean = 18.49<br>College students			C: Anagram task<br>Correction number↑	
3	Graham et al. (2014)	Stroop task	5min	72<br>Sex: 23♂; 49♀<br>Age: 22.33±3.46	*Autonomy-supportive*<br>*Motivation:* autonomous regulation - performing an action that one freely chooses,	(i) The resource model of self-	P: Endurance Trial<br>Persistence time↑	Exercises

				University students	or is supported to do, for the inherent satisfaction.<br><br>Instruct subjects: "how much effort you put into this task is ultimately up to you".	regulation<br><br>(ii) Self-determination theory		
4	Zhu et al. (2017)	Cross-off-the-letter task	UA	63<br>Sex: 17♂; 46♀<br>Age: 21.56± 2.41<br>University students	*Motivation:* Motivation is an inner incentive that compels individuals to attain certain goals and sustain goal-directed behaviors.<br><br>After the mental exertion, subjects were told that they could receive monetary rewards in different level of persistence time.	The resource model of self-regulation	P: Proximal dumbbell task<br>Persistence time↑<br>Distal dumbbell task<br>Persistence time ↔	Social behavior
5	Brown and Bray (2017)	Stroop task	10min	82<br>Sex: 30♂; 52♀<br>Age: 20.02± 1.52<br>university students	*Motivation:* is now considered a major factor in cognitive control and behavior. Motivated people can further draw upon self-control resources.<br><br>Subjects would earn extra money if they matched or exceeded their performance from the first handgrip endurance trial.	The resource model of self-regulation	P: Handgrip task<br>Persistence time↑	Exercise

C: cognitive task; P: physical task; UA: unavailable; ♀: female; ♂: male.

Allom, V., & Mullan, B. (2015). Two inhibitory control training interventions designed to improve eating behaviour and determine mechanisms of change. *Appetite*, *89*, 282-290. https://doi.org/10.1016/j.appet.2015.02.022

Axelsen, J. L., Kirk, U., & Staiano, W. (2020). On-the-spot binaural beats and mindfulness reduces the effect of mental fatigue. *Journal of Cognitive Enhancement*, *4*(1), 31-39. https://doi.org/10.1007/s41465-019-00162-3

Bennett, M. (2019). The effect of sound on attention restoration. *Senior Honors Theses*, 267. https://digitalcommons.brockport.edu/honors/267

Berman, M. G., Jonides, J., & Kaplan, S. (2008). 50. The Cognitive Benefits of Interacting With Nature. *Psychological Science*, *19*(12), 1207-1212. https://doi.org/10.1111/j.1467-9280.2008.02225.x

Berto, R. (2005). Exposure to restorative environments helps restore attentional capacity. *Journal of Environmental Psychology*, *25*(3), 249-259. https://doi.org/10.1016/j.jenvp.2005.07.001

Bertrams, A., & Schmeichel, B. J. (2014). Improving self-control by practicing logical reasoning. *Self and Identity*, *13*(4), 419-431. https://doi.org/10.1080/15298868.2013.836562

Beute, F., & de Kort, Y. A. W. (2014). 50. Natural resistance: Exposure to nature and self-regulation, mood, and physiology after ego-depletion. *Journal of Environmental Psychology*, *40*, 167-178. https://doi.org/10.1016/j.jenvp.2014.06.004

Boucher, H. C., & Kofos, M. N. (2012). 50. The idea of money counteracts ego depletion effects. *Journal of Experimental Social Psychology*, *48*(4), 804-810. https://doi.org/10.1016/j.jesp.2012.02.003

Bray, S. R., Graham, J. D., & Saville, P. D. (2015). Self-control training leads to enhanced cardiovascular exercise performance. *Journal of Sports Sciences*, *33*(5), 534-543. https://doi.org/10.1080/02640414.2014.949830

Brown, D. M. Y., & Bray, S. R. (2017). 50. Effects of Mental Fatigue on Physical Endurance Performance and Muscle Activation Are Attenuated by Monetary Incentives. *Journal of Sport & Exercise Psychology*, *39*(6), 385-396. https://doi.org/10.1123/jsep.2017-0187

Brown, D. M. Y., & Bray, S. R. (2019). 50. Heart rate biofeedback attenuates effects of mental fatigue on exercise performance. *Psychology of Sport and Exercise*, *41*, 70-79. https://doi.org/10.1016/j.psychsport.2018.12.001

Chow, J. T., & Lau, S. (2015). 50. Nature Gives Us Strength: Exposure to Nature Counteracts Ego-Depletion. *Journal of Social Psychology*, *155*(1), 70-85. https://doi.org/10.1080/00224545.2014.972310

Cranwell, J., Benford, S., Houghton, R. J., Golembewski, M., Fischer, J. E., & Hagger, M. S. (2014). Increasing self-regulatory energy using an Internet-based training application delivered by smartphone technology. *Cyberpsychol Behav Soc Netw*, *17*(3), 181-186. https://doi.org/10.1089/cyber.2013.0105

Dang, J., Xiao, S., Shi, Y., & Mao, L. (2015). 在 mental exertion 之前 CP Action orientation overcomes the ego depletion effect. *Scandinavian Journal of Psychology*, *56*(2), 223-227. https://doi.org/10.1111/sjop.12184

Denson, T. F., Capper, M. M., Oaten, M., Friese, M., & Schofield, T. P. (2011). Self-control training decreases aggression in response to provocation in aggressive individuals. *Journal of Research in Personality*, *45*(2), 252-256. https://doi.org/10.1016/j.jrp.2011.02.001

Echo Wen, W., & Sternthal, B. (2008). Regulating the Effects of Depletion Through Monitoring. *Personality and Social Psychology Bulletin*, *34*(1), 32-46. https://doi.org/10.1177/0146167207306756

Emfield, A. G., & Neider, M. B. (2014). Evaluating visual and auditory contributions to the cognitive restoration effect. *Frontiers in Psychology*, *5*(548 %M), %7 %8 2014-June-2005 %2019 Original Research %+ Dr Mark B. Neider,mark.neider@ucf.edu %# %! EVALUATING CONTRIBUTIONS TO COGNITIVE RESTORATION %* %<.

https://doi.org/10.3389/fpsyg.2014.00548 %W %L

Evensen, K. H., Raanaas, R. K., Hagerhall, C. M., Johansson, M., & Patil, G. G. (2015). Restorative elements at the computer workstation: A comparison of live plants and inanimate objects with and without window view. *Environment and Behavior*, *47*(3), 288-303. https://doi.org/10.1177/0013916513499584

Filipas, L., Martin, K., Northey, J. M., La Torre, A., Keegan, R., & Rattray, B. (2020). 57. A 4-week endurance training program improves tolerance to mental exertion in untrained individuals. *Journal of Science and Medicine in Sport*, *23*(12), 1215-1219. https://doi.org/10.1016/j.jsams.2020.04.020

Friese, M., Messner, C., & Schaffner, Y. (2012). Mindfulness meditation counteracts self-control depletion. *Consciousness and Cognition*, *21*(2), 1016-1022. https://doi.org/10.1016/j.concog.2012.01.008

Gailliot, M. T., Plant, E. A., Butz, D. A., & Baumeister, R. F. (2007). Increasing self-regulatory strength can reduce the depleting effect of suppressing stereotypes. *Personality and Social Psychology Bulletin*, *33*(2), 281-294. https://doi.org/10.1177/0146167206296101

Graham, J. D., Bray, S. R., & Martin Ginis, K. A. (2014). "Pay the piper": It helps initially, but motivation takes a toll on self-control. *Psychology of Sport and Exercise*, *15*(1), 89-96. https://doi.org/10.1016/j.psychsport.2013.09.007

Haga, A., Halin, N., Holmgren, M., & Sörqvist, P. (2016). Psychological Restoration Can Depend on Stimulus-Source Attribution: A Challenge for the Evolutionary Account? . *Frontiers in Psychology*, *7*(1831 %M), %7 %8 2016-November-2023 %2019 Original Research %+ Patrik Sörqvist,Department of Building, Energy and Environmental Engineering, University of Gävle,Gävle, Sweden,patrik.sorqvist@hig.se %# %! RESTORATION CAN DEPEND ON SOURCE ATTRIBUTION %* %<. https://doi.org/10.3389/fpsyg.2016.01831 %W %L

Laumann, K., Gärling, T., & Stormark, K. M. (2003). Selective attention and heart rate responses to natural and urban environments. *Journal of Environmental Psychology*, *23*(2), 125-134. https://doi.org/https://doi.org/10.1016/S0272-4944(02)00110-X

Lee, K. E., Williams, K. J. H., Sargent, L. D., Williams, N. S. G., & Johnson, K. A. (2015). 40-second green roof views sustain attention: The role of micro-breaks in attention restoration. *Journal of Environmental Psychology*, *42*, 182-189. https://doi.org/10.1016/j.jenvp.2015.04.003

Lobo, A., Ramachandran, H., & Tiwari, A. (2015). The effect of viewing nature and urban pictures on affect and cognition. *Journal of the Indian Academy of Applied Psychology*, *41*(Special Issue 3), 162-174.

Loch, F., Hof zum Berge, A., Ferrauti, A., Meyer, T., Pfeiffer, M., & Kellmann, M. (2020). 49. Acute Effects of Mental Recovery Strategies After a Mentally Fatiguing Task. *Frontiers in Psychology*, *11*, Article 558856. https://doi.org/10.3389/fpsyg.2020.558856

Miles, E., Sheeran, P., Baird, H., Macdonald, I., Webb, T. L., & Harris, P. R. (2016). Does Self-Control Improve With Practice? Evidence From a Six-Week Training Program. *Journal of Experimental Psychology-General*, *145*(8). https://doi.org/10.1037/xge0000185

Muraven, M., Baumeister, R. F., & Tice, D. M. (1999). Longitudinal Improvement of Self-Regulation Through Practice. *The Journal of Social Psychology*, *42*(2), 130-

153. https://doi.org/10.1086/250095

Muraven, M., & Slessareva, E. (2003). Mechanisms of Self-Control Failure: Motivation and Limited Resources. *Personality and Social Psychology Bulletin*, *29*(7), 894-906. https://doi.org/10.1177/0146167203029007008

Neilson, B. N., Craig, C. M., Curiel, R. Y., & Klein, M. I. (2020). Restoring Attentional Resources With Nature: A Replication Study of Berto's (2005) Paradigm Including Commentary From Dr. Rita Berto. *Human Factors*, 0018720820909287. https://doi.org/10.1177/0018720820909287

Oaten, M., & Cheng, K. (2006a). Improved self-control: the benefits of a regular program of academic study. *Terapevticheskii Arkhiv*, *65*(11), 22-25.

Oaten, M., & Cheng, K. (2006b). Longitudinal gains in self-regulation from regular physical exercise. *British Journal of Health Psychology*, *11*(4), 717-733. https://doi.org/10.1348/135910706X96481

Oaten, M., & Cheng, K. (2007). Improvements in self-control from financial monitoring. *Journal of Economic Psychology*, *28*(4), 487-501. https://doi.org/10.1016/j.joep.2006.11.003

Pilotti, M., Klein, E., Golem, D., Piepenbrink, E., & Kaplan, K. (2015). Is viewing a nature video after work restorative? Effects on blood pressure, task performance, and long-term memory. *Environment and Behavior*, *47*(9), 947-969. https://doi.org/10.1177/0013916514533187

Shaabani, F., Naderi, A., Borella, E., & Calmeiro, L. (2020). 30.Does a Brief Mindfulness Intervention Counteract the Detrimental Effects of Ego Depletion in Basketball Free Throw Under Pressure? *Sport Exercise and Performance Psychology*, *9*(2), 197-215. https://doi.org/10.1037/spy0000201

Stocker, E., Englert, C., & Seiler, R. (2019). 27. Self-Control Strength and Mindfulness in Physical Exercise Performance: Does a Short Mindfulness Induction Compensate for the Detrimental Ego Depletion Effect? *Journal of Applied Sport Psychology*, *31*(3), 324-339. https://doi.org/10.1080/10413200.2018.1471754

Tyler, J. M., & Burns, K. C. (2008). After depletion: The replenishment of the self's regulatory resources. *Self and Identity*, *7*(3), 305-321. https://doi.org/10.1080/15298860701799997

Valtchanov, D., Barton, K. R., & Ellard, C. (2010). 没有 C P***Restorative effects of virtual nature settings. *Cyberpsychology, Behavior, and Social Networking*, *13*(5), 503-512. https://doi.org/10.1089/cyber.2009.0308

Webb, T. L., & Sheeran, P. (2003). 16. Can implementation intentions help to overcome ego-depletion? *Journal of Experimental Social Psychology*, *39*(3), 279-286. https://doi.org/10.1016/s0022-1031(02)00527-9

Zhang, Y., Kang, J., & Kang, J. (2017). Effects of Soundscape on the Environmental Restoration in Urban Natural Environments. *Noise & Health*, *87*(19).

Zhu, Z., Li, J., Zhang, B., Li, Y., & Zhang, H. (2017). 4. The effect of motivation and positive affect on ego depletion: Replenishment versus release mechanism. *International Journal of Psychology*, *52*(6), 445-452. https://doi.org/10.1002/ijop.12235
